# Role of the gut microbiota and their metabolites in hemodialysis patients

**DOI:** 10.7150/ijms.82667

**Published:** 2023-04-17

**Authors:** Ying Ting Chao, Ying-Kuang Lin, Liang-Kun Chen, Poyin Huang, Yi-Chiung Hsu

**Affiliations:** 1 Department of Biomedical Sciences and Engineering, National Central University, Taoyuan 320317, Taiwan, R.O.C.; 2 Division of Nephrology, Department of Medicine, Landseed International Hospital, Taoyuan City 324609, Taiwan, R.O.C.; 3 Department of Neurology, Kaohsiung Medical University Hospital, Kaohsiung Medical University, Kaohsiung City, 807, Taiwan, R.O.C.; 4 Department of Neurology, Kaohsiung Municipal Siaogang Hospital, Kaohsiung Medical University, Kaohsiung City, 807, Taiwan R.O.C.; 5 Neuroscience Research Center, Kaohsiung Medical University, Kaohsiung City, 807, Taiwan R.O.C.; 6 Department of Neurology, Faculty of Medicine, College of Medicine, Kaohsiung Medical University, Kaohsiung City, 807, Taiwan R.O.C.

**Keywords:** calcium-phosphorus product, chronic kidney disease, gut microbiota, dysbiosis

## Abstract

High serum phosphate levels in chronic kidney disease (CKD) are linked to adverse health outcomes, including cardiovascular disease, kidney disease progression, and all-cause mortality. This study is aimed to find out which microorganisms or microbial functions have a significant impact on higher calcium-phosphorus product (Ca x P) after they undergo hemodialysis (HD) treatment. *Feces* samples from 30 healthy controls, 15 dialysis patients with controlled Ca xP (HD), and 16 dialysis patients with higher Ca xP (HDHCP) were collected to perform in 16S amplicon sequencing.

We found gut microbial composition was significantly different between hemodialysis patients and healthy controls. Three phyla including *Firmicutes*, *Actinobacteria*, and *Proteobacteria* were significantly enriched in hemodialysis patients. Although only one genus, *Lachnospiraceae_FCS020_group*, was significantly increased in higher Ca xP group, there were four metabolic pathways predicted by PICRUSt significantly increased in higher Ca xP group and associated with causing VC, including the pentose phosphate pathway, steroid biosynthesis, terpenoid backbone biosynthesis, and fatty acid elongation pathway. Characterizing dysbiosis of gut microbiome played the important role in hemodialysis patients.

## Introduction

Chronic kidney disease (CKD) is one of the leading causes of death globally in the 21^st^ century and affects 10% of the general population worldwide, amounting to more than 800 million individuals 1. Cardiovascular disease (CVD) is the main cause of morbidity and mortality among patients with CKD 2, 3, and the mortality of CVD patients with chronic renal disease was higher. The mortality rate of the patients who *undergo hemodialysis* (HD), and peritoneal *dialysis* (PD) are 10 to 20 times than the general population 4. Furthermore, vascular calcification (VC) is a risk factor associated with major adverse cardiovascular events 5. Vascular calcification is linked to elevated calcium-phosphorus product (Ca × P) [6.7]. End-stage renal failure, also known as end-stage renal disease (ESRD), occurs when chronic kidney disease. The kidney of ESRD patients lost filtering abilities can no longer function on their own.

A ESRD patient must receive HD, PD or kidney transplantation in order to survive. Kraus et al. demonstrated that 100% of patients withESRD on the treatment with hemodialysis for 3 months suffer from calcification in the aortic valve, mitral valve, or mitral annulus by echocardiography, and 77.8% of X-rayed patients suffer from abdominal aortic calcification 8. In those CKD patients who haven't applied dialysis, the incidence rate of coronary artery calcification is 25.5% 9.

VC was also induced by high phosphorus caused by CKD, parathyroid hormone (PTH), inflammatory cytokines, oxidative stress, and uremic toxins 10. Moreover, the gut microbiota-derived metabolites like p-cresol (pCS), indoxyl sulfate (IS), Trimethylamine N-oxide (TMAO), bile acid, and phenylacetylglutamine affected vascular smooth muscle cells or vascular endothelial cells which directly or indirectly induce VC 10, 11. The human gut microbiota collectively made up to 100 trillion archaeal and bacterial cells and contained over 99% of bacteria distributed over more than 1,000 species 12. The relative abundance of major bacterial populations in fecal samples from patients with CKD and patients with ESRD versus healthy controls was shifted 11. Much direct experimental evidence supported the contribution of fecal microbial community changes in CVD 13. Therefore, we mainly focus on microorganisms or microbial functions which may be contributing to the increased risk of higher calcium-phosphorus product (Ca × P) by discovering gut microbial profiles between healthy controls, and hemodialysis patients with or without higher Ca × P.

## Materials and methods

### Patients and Controls

The hemodialysis or with higher Ca × P patients who signed informed consent and would be compliant for follow-up were recruited to the study. The study conformed to the ethical guidelines of the 1975 Declaration of Helsinki and was approved by the Institutional Review Board of the [IRB-20-046-B1]. The recruited patients will be collected their basic demographics, hospital courses, use of antibiotics, type of feedings, and other treatments in the Landseed International Hospital. The patients we collected in the study have been excluded if they use either antibiotics or probiotics. We used calcium-phosphate product (Ca x P) as an indicator which is a clinically relevant tool to estimate the risk of patients with renal failure. The calcium phosphorus product must be kept <55 mg^2^ / dL^2^ 6. CKD patients are controlled their metabolism by monitoring the plasma levels of phosphorus within the range of target values. When the score of calcium-phosphate product is greater than 60 mg^2^ / dL^2^, we employed these cut-off values to identify patients with higher Ca x P levels.

Both patients on hemodialysis and control subjects were asked to bring a fecal sample to the laboratory within 12 h of defecation. After collection of the fecal sample, an aliquot was immediately frozen and stored at -20°C until further analysis.

### Stool DNA extraction and 16S rRNA gene amplification

DNA was extracted with QIAamp Fast DNA Stool Mini Kit (Qiagen, Hilden, Germany) all according to respective manufacturers' instructions.

The 16S rRNA sequencing libraries are followed according to the manufacturer's instructions provided by Illumina (Illumina, CA, USA). Briefly, 12.5 ng of DNA is used for PCR amplification of the V3 and V4 regions of 16S rRNA gene. The PCR products are purified with AMPure XP beads (Beckman Coulter, USA) and subjected to a secondary PCR reaction with primers from Nextera XT Index kit (Illumina, CA, USA) by adding dual indices and Illumina sequencing adapters. After PCR reaction, the final libraries (~630 bp) are purified with AMPure XP beads and ready for next-generation sequencing.

### 16S rRNA Gene Amplicon Sequencing

The concentrations of 16S rRNA sequencing libraries are determined by real-time quantitative PCR with Illumina adapter-specific primers provided by KAPA library quantification kit (KAPA Biosystems, USA). Libraries are denatured and sequenced by Illumina MiSeq platform with reagent v3 for pair-end sequencing (2*250 bp/ 2*300 bp). Instrument control, cluster generation, image capture and base calling are processed by Real-Time Analysis software (RTA), MiSeq Control software (MCS) and MiSeq Report software (MSR) on MiSeq platform. FASTQ files generated by MiSeq Report are used for further analysis.

### Bioinformatics and statistical analysis

Raw reads were processed using the mothur 14 V1.35.0 and were based upon a protocol developed for MiSeq by the mothur creators 15 with default parameters. The read pairs were assembled into contigs by aligning the reads and any sequences which failed to align were discarded. The sequence was then aligned to SILVA reference alignment 16 and any reads aligned outside of the V3-V4 region were removed from the dataset. Chimeras were identified and removes using UCHIME 17. The sequences were classified using mothur's Bayesian classifier against the SILVA database (release 128) 16. Sequences identified as chloroplast, mitochondria or not originating from bacteria were removed from further analysis. The sequences were then clustered into Operational Taxonomic Units (OTUs) based on 97% sequence similarity. Taxa summaries, OTU heatmap and alpha rarefaction were performed in QIIME 18. Principal coordinate analyses (PCoA) were based on unweighted and weighted UniFrac distances and calculated in QIIME. Non-metric multidimensional scaling (NMDS) and canonical correspondence analysis (CCA) were analyzed using vegan (R package: v2.4 1) 19 package and heatmap were analyzed using gplots 20 package in R. High-dimensional biomarker discovery and explanation that identifies genomics taxonomy characterizing the differences between two or more biological conditions were performed in LEfSe v1.0 21. Differential analyses of the count data at the OTU level were applied to use negative binomial generalized linear model implemented in DESeq2 22, 23 R package. Functional contents of the 16S rRNA were predicted using the PICRUSt software v2.2.0 b 24. Differential analyses of the functional contents were also using negative binomial generalized linear model implemented in DESeq2 R package. The statistical significance related to the relative abundance was using one‐way ANOVA and post‐hoc Tukey's test, GraphPad Prism® version 9 (GraphPad Software, USA).

## Results

### Clinical characteristics of the Participants

A total of 61 stool samples were collected, including 30 healthy controls (Ctrl), 15 hemodialysis patients without higher Ca x P (HD), and 16 hemodialysis patients with higher Ca x P (HDHCP).

Clinical characteristics were described in Table 1. In the validation cohort, the proportions of women and men were similar in each group. The average age of Ctrl, HD and HDHCP patients were at age 58.3, 63.5 and 55.3. Hemodialysis time was not significantly different between HD and HDHCP. However, in HD group, serum levels of creatinine (Cr), uric acid (UA), albumin (Alb), sodium (Na), calcium-phosphate product (Ca×P), calcium (Ca) and Intact Parathyroid hormone (iPTH) were significantly increased in HDHCP (p < 0.05).

### Gut microbial diversity of healthy controls and hemodialysis patients

A total of 19,647,778 sequencing sequences were generated from stool samples collected from 61 participants (range, 166,892- 772,186). After quality and size filtered, and host sequence removed, 15,326,912 reads were clustered into OTUs (range, 123,032- 715,042). A total of 100,8282 OTUs with more than 1 read were generated for downstream analysis. Rarefaction analysis showed that the number of OTUs was significantly increased in hemodialysis patients (n = 31) compared to that in healthy controls (n = 30) (p < 0.0001) (Supplementary data S1 A). As measured by the Chao index (Supplementary data S1 B), the Ace index (Supplementary data S1 C), and the Shannon index (Supplementary data S1 D), the gut microbial diversity was significantly increased in HD and HDHCP compared to Ctrl (p ≦ 0.0001).

**Figure 1:**
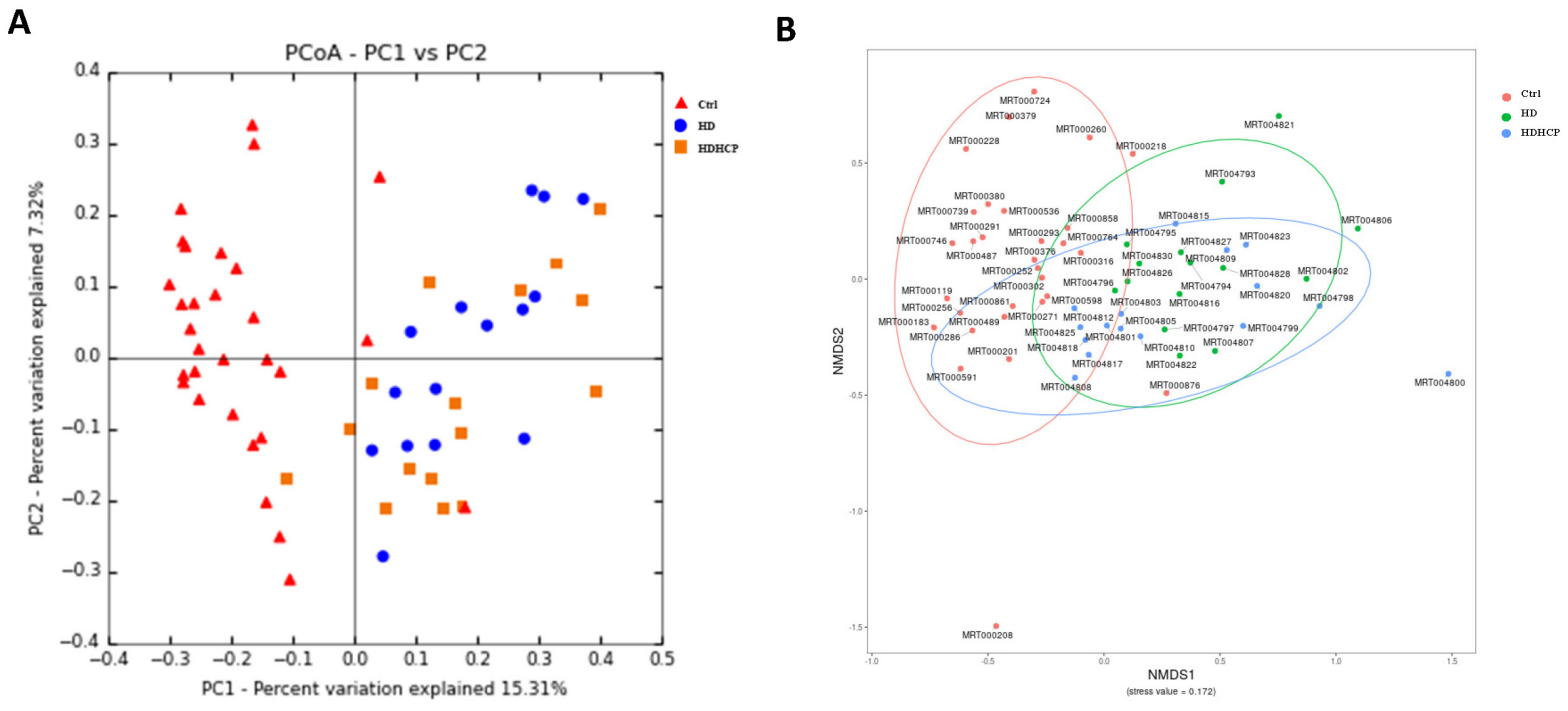
Gut microbial diversity based on OTUs distribution in healthy controls and hemodialysis patients (A-B).

In addition, the principal coordinate analysis (PCoA), non-metric multidimensional scaling (NMDS) and the statistical analysis of Adonis based on OTUs distribution were executed to illustrate the diversity. The gut microbial composition was significantly different between hemodialysis patients and healthy controls (Figure 1A and 1B). While diversity in hemodialysis patients with higher Ca x P was not different significantly from hemodialysis patients without higher Ca x P.

### Gut microbiome in healthy controls and hemodialysis patients

**Figure 2:**
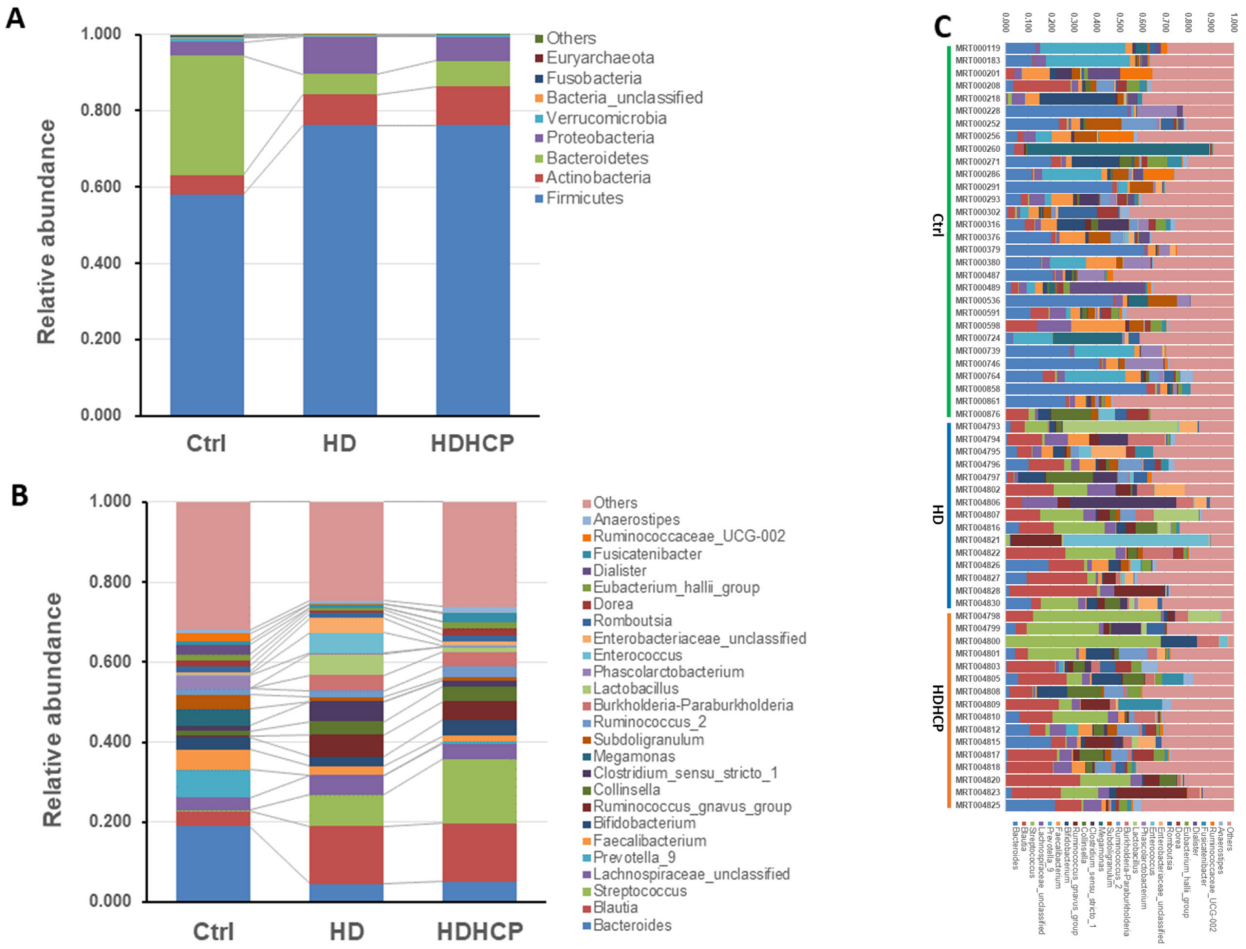
Gut microbial composition of healthy controls and hemodialysis patients. Average compositions and relative abundance of the bacterial community in three groups at levels of the phylum (A) and the genus (B). (C) The difference in gut microbial composition was shown in Ctrl, HD and HDHCP.

Further analysis is to do taxonomic analysis. Average compositions and relative abundance of the bacterial community in three groups at the phylum and the genus levels have been shown in Figure 2A and 2B. Three phyla including *Firmicutes*, *Actinobacteria* and *Proteobacteria* were significantly enriched, while *Bacteroidetes* and *Verrucomicrobia* were significantly decreased in hemodialysis patients.

At the genus level, *Blautia*, *Streptococcus*,* Lachnospiraceae_unclassified*, *Ruminococcus_gnavus_group*, *Collinsella* and *Burkholderia-Paraburkholderia* in hemodialysis patients are the most abundant and more than that in the healthy controls. The difference in gut microbial composition was shown in Ctrl, HD and HDHCP (Figure 2C).

### Potential bacteria related to higher Ca x P in hemodialysis patients

**Figure 3:**
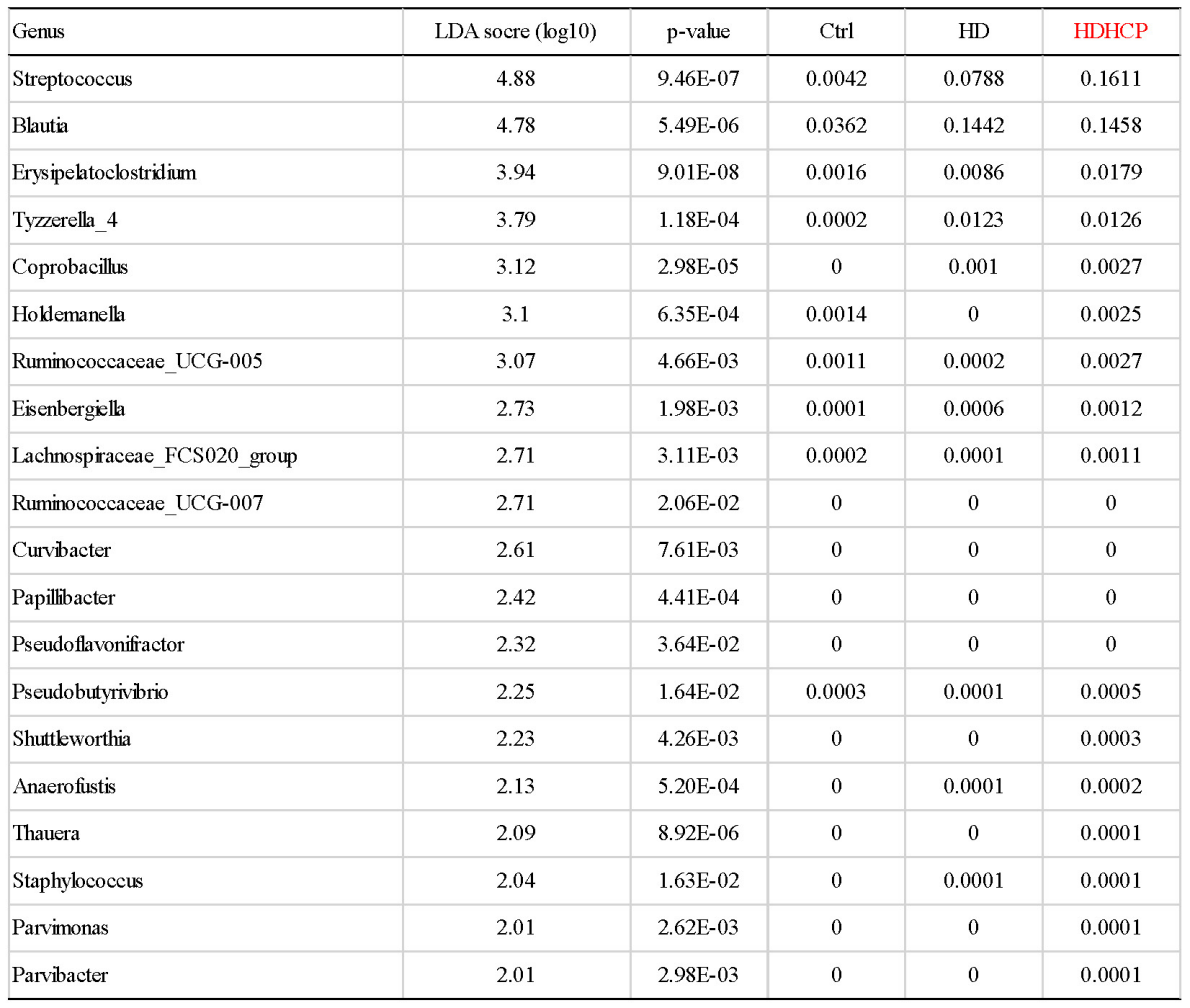

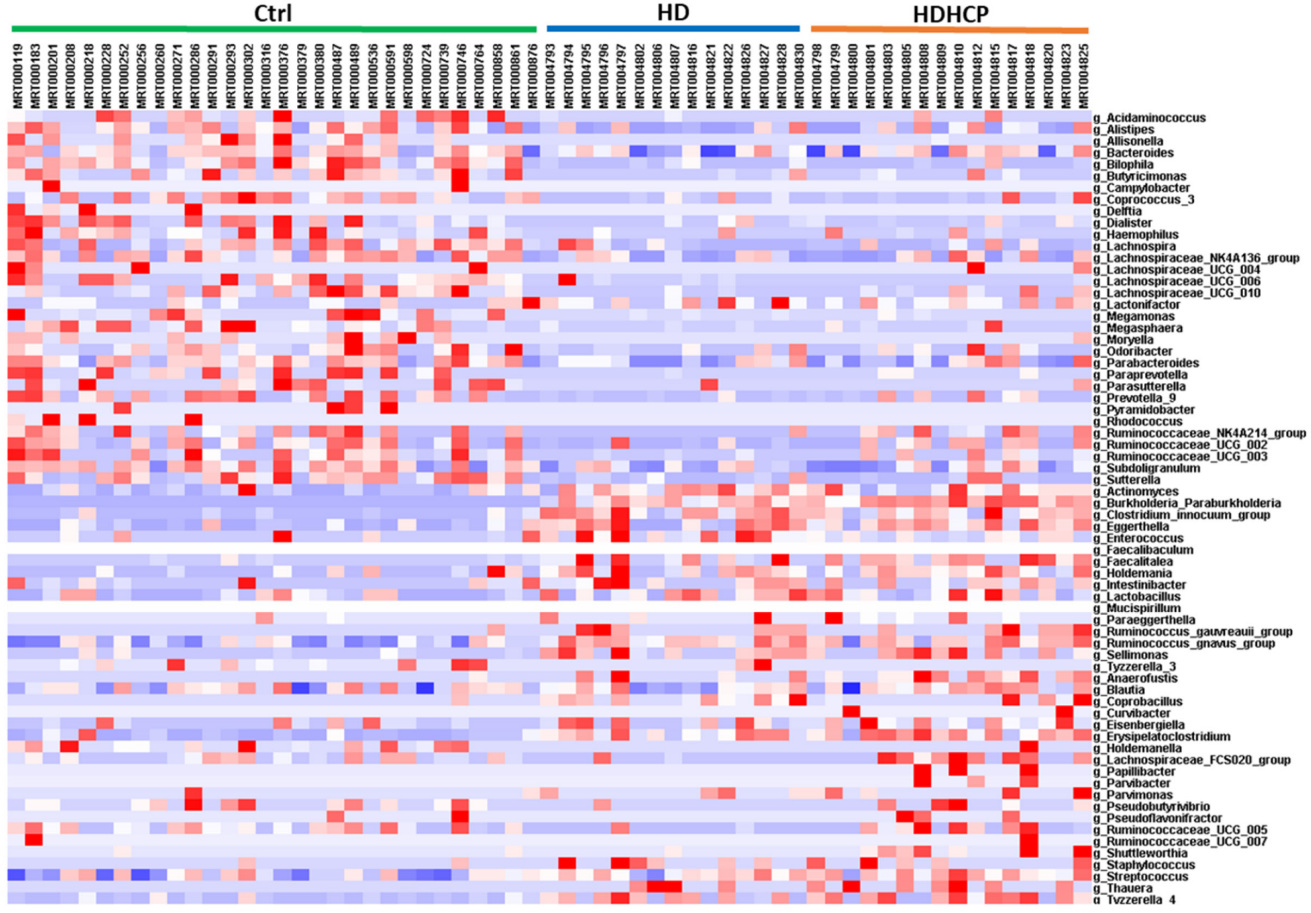
Heatmap showing the relative abundance of the 68 genera that were significantly enriched between Ctrl, HD, and HDHCP.

Linear discriminant analysis effect size (LEfSe) was used to compare the presence and impact of region-specific OTUs in three groups, to determine the specific bacterial taxa and predominant. The threshold of the linear discriminant analysis (LDA) was >2.0 and p<0.05. The relative abundances of the family *Streptococcaceae*, *Coriobacteriaceae, Erysipelotrichaceae*, *Dermabacteraceae*, Dermatophilaceae, Micrococcaceae, Family_XI and *Rhodocyclaceae* were found to be higher in HDHCP. The taxonomic clusters based on LDA selection at the genus level were shown in Supplementary data S2. According to the LDA selection, 32 genera were significantly enriched in Ctrl, 16 genera in HD and 20 genera in HDHCP. Heatmap analysis of the relative abundance of the 68 genera was demonstrated the LEfSe result (Figure 3). In particular, the relative abundances of the genera *Streptococcus*, *Blautia*, *Erysipelatoclostridium*, *Tyzzerella_4*, *Coprobacillus*, *Holdemanella*, *Ruminococcaceae_UCG-005*, *Eisenbergiella Lachnospiraceae_FCS020_group*, *Ruminococcaceae_UCG-007*, *Curvibacter*, *Papillibacter*, *Pseudoflavonifractor*, *Pseudobutyrivibrio*, *Shuttleworthia*, *Anaerofustis*, *Thauera*, *Staphylococcus*, *Parvimonas* and *Parvibacte*r were higher in HDHCP (Table 2).

**Figure 4:**
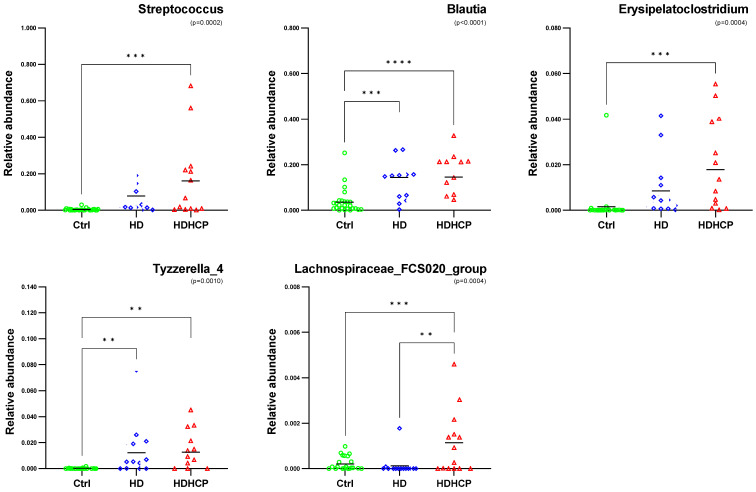
Relative abundance of 5 genera with significantly enriched in HDHCP and average abundance more than 0.001 in Ctrl, HD and HDHCP.

One-way ANOVA was used to analyze the statistical significance of 20 genera significantly enriched in HDHCP. As the result showed in Figure 4, 5 genera including *Streptococcus*, *Blautia*, *Erysipelatoclostridium*, *Tyzzerella_4* and *Lachnospiraceae_FCS020_group* were significantobserved in HDHCP. Besides, only the *Lachnospiraceae_FCS020_group* was significantly enriched in HDHCP compared to HD.

### Correlation between the Gut Microbiome and Clinical Indicators of hemodialysis patients

**Figure 5:**
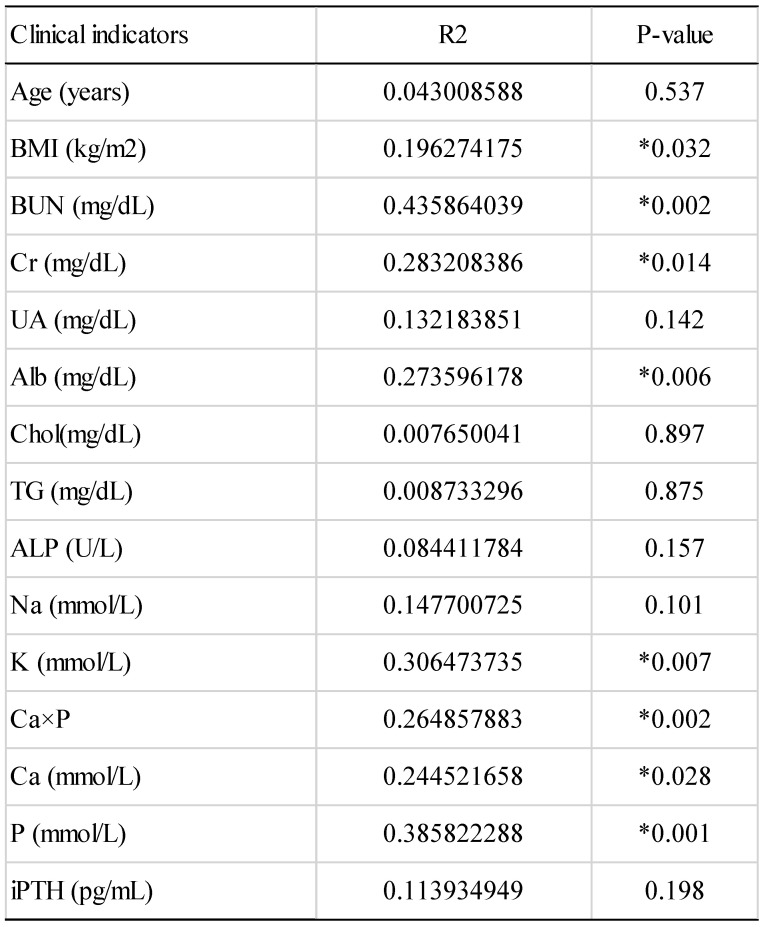

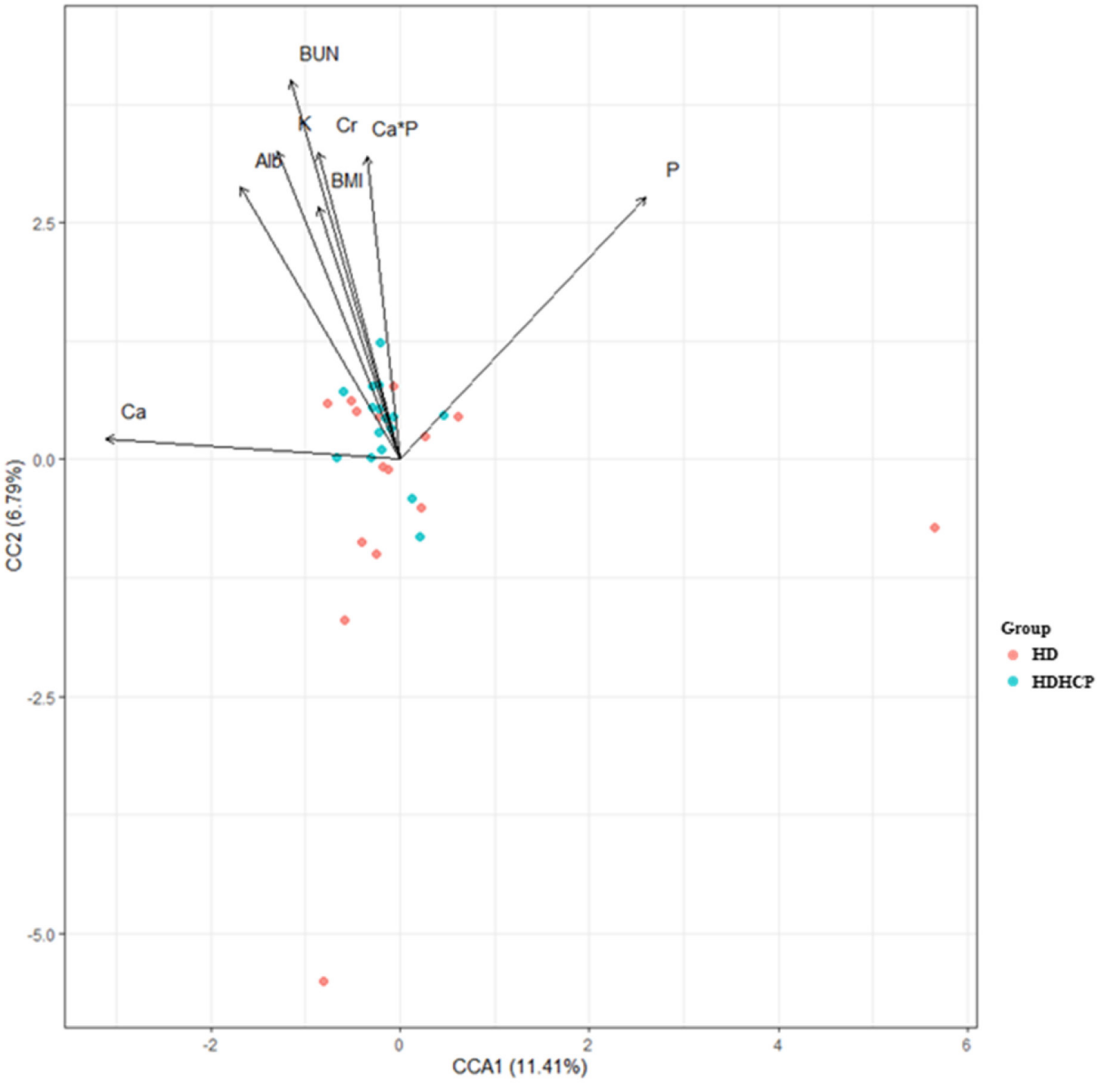
The CCA analysis of the associations between the gut microbiome and clinical indicators for hemodialysis patients in the HD and HDHCP.

To further understand the impact of clinical indicators of hemodialysis patients on the gut microbiome in the HD and HDHCP, canonical correspondence analysis (CCA) was performed at the OTU level. Fifteen clinical indicators of hemodialysis patients (Age, BMI, BUN, Cr, UA, Alb, Chol, TG, ALP, Na, K, (Ca×P), Ca, P and iPTH) were analyzed. CCA of the gut microbiome and these clinical indicators were shown in Table 3. Eight clinical indicators (BMI, BUN, Cr, Alb, K, Ca*P, Ca and P) were closely related to the gut microbiome in hemodialysis patients (Figure 5).

### Crucial microbial functions related to higher Ca x P in dialysis patients

**Figure 6:**
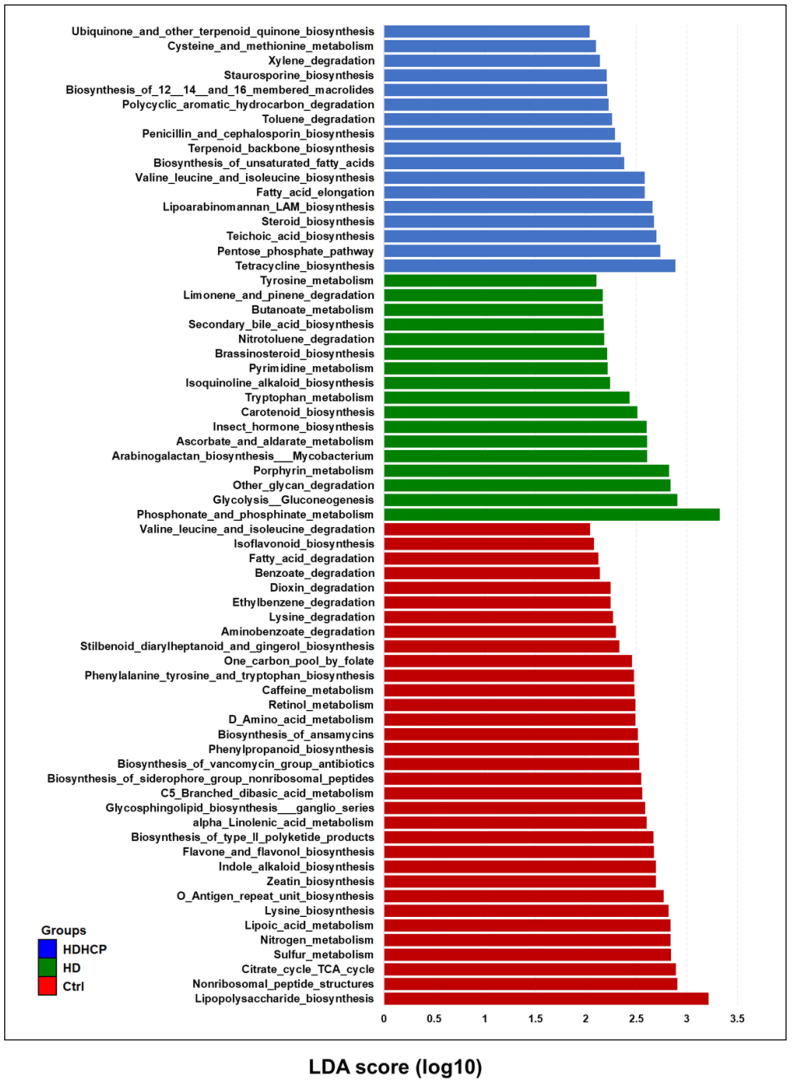
Functional alterations of the gut microbiome in healthy controls and hemodialysis patients. LEfSe results showed a statistically significant increase in the abundance metabolism of KEGG pathways in the Ctrl, HD, and HDHCP.

PICRUSt software was used to predict the relative abundance of functions according to the obtained 16S rRNA sequences to determine the abundance and enrichment of functional genes at different levels in the Kyoto Encyclopedia of Genes and Genomes (KEGG) pathways. The predicted functions classified as metabolism at the first level of the KEGG were conducted to identify the predominant microbial functions by LEfSe. According to the LDA selection, 67 predicted microbial functions were shown in Figure 6. The result exhibited 17 functions including tryptophan metabolism, tyrosine metabolism and secondary bile acid biosynthesis were increased in HD. In addition, 17 functions including pentose phosphate pathway (PPP), steroid biosynthesis, terpenoid backbone biosynthesis, and fatty acid elongation pathway were increased in HDHCP.

## Discussion

*Two dominant bacterial phyla*, *Bacteroidetes* and *Firmicutes*, were found in more than 90% of the gut microbiome of healthy people 26. The total percentage of *Bacteroidetes* and *Firmicutes* of the gut microbiome was almost 90% in the healthy controls, but only 81.6% and 82.9% in HD and HDHCP, respectively. The proportion changed was affected by the decrease of *Bacteroidetes* in the gut microbiome of hemodialysis patients (Ctrl:31.2%, HD:5.5% and HDHCP: 6,7%) (Supplementary data S3). According to Bao et al. study, their study purpose is similar with us 27. They indicated the majority of phyla in the gut microbiome of hemodialysis (HD) patients were *Firmicutes, Actinobacteriota, Proteobacteria, and Bacteroidota* which had same trend compared to our results. In terms of abundant phyla, our result was consistent with theirs. The percentages in our result of these two phyla *Firmicutes* and *Actinobacteriota* were over 70% and 8-10% respectively in HD patients, which the proportion is 70% and 10% in their study. In Ana Merino-Ribas' study, they included 44 CKD patients undergoing peritoneal dialysis 28. The results of the gut microbiome at the phylum level enriched in *Firmicutes* and *Bacteroidota* which were consistent with us. However, the abundance composition ratio in PD patients was not in consonance with our study.

The decrease of protein digestion and absorption capacity occurring in CKD patients when the decrease of fiber intake and the increase of protein intake in the dietary treatment, leads to the decrease of short-chain fatty acids and the increase of protein in the gut, which in turn causes their dysbiosis 29. Shin et al proposed that an increased prevalence of* Proteobacteria* would be a potential diagnostic signature of dysbiosis and risk of disease due to an imbalanced gut microbiota often arose from a sustained increase in abundance of *Proteobacteria* 30. The content of *Proteobacteria* in the gut microbiome of healthy people was less than 1% 26. While the relative abundance of *Proteobacteria* was increased more significantly in hemodialysis patients than healthy controls. This result was concordant with other studies 31, 32 which is a characterization of dysbiosis in CKD patients.

The diversity of the gut microbiome in heathy controls was higher than mild CKD and moderate CKD patients, but lower than advanced CKD patients 32. Our result also demonstrated that the diversity involved both taxon richness and evenness in hemodialysis patients was significantly higher than healthy controls. Nevertheless, the diversity involved both taxon richness and evenness were similar as estimated by observed species, Chao1, Ace, Shannon diversity indexes between HD and HDHCP. Similarly, very few bacteria were showed significant differences between HD and HDHCP. Among the 65 genera selected by LDA, only *Lachnospiraceae_FCS020_group* was significantly different between HD and HDHCP.

*Lachnospiraceae_FCS020_group* was a genus of *Lachnospiraceae* family producing short chain fatty acids (SCFA). TMAO induced inflammatory gene expression on vascular smooth muscle cells to contribute to atherosclerosis and is associated with coronary artery disease risk 33. While serum TMAO levels were positively associated with decreased abundance of *Lachnospiraceae_FCS020_group* 34. In addition,* Lachnospiraceae_FCS020_group* was found to be negatively associated with triglycerides in VLDL particles of various sizes, small HDL particles, and medium HDL particles 35. According to these research data, *Lachnospiraceae_FCS020_group* seemed to be negatively associated with VC, but it was probably due to the *insufficient* published data.

pCS, IS, TMAO, bile acid and phenylacetylglutamine induced VC were derived from gut microbiome 10, 11. pCS, IS and bile acid originated from tyrosine metabolism, tryptophan metabolism and secondary bile acid biosynthesis, respectively. All three metabolic pathways were significantly increased in HD, but not in HDHCP. Although the blood vessels did not show calcification in HD, the three metabolic pathways were significantly increased. This indicated that the production of pCS, IS and bile acid probably caused the blood *vascular* abnormalities and raised the risk of VC. In HDHCP group, the metabolic pathways which significantly increased and potentially causing VC were PPP, steroid biosynthesis, terpenoid backbone biosynthesis, and fatty acid elongation pathway.

The PPP was related to the regulation of the intracellular Ca^2+^. The inhibition of PPP decreased intracellular Ca^2+^ to oxidize NADPH and GSH and appears to activate a novel coordination of redox-controlled relaxing mechanisms in bovine coronary arteries 36. Peiró et al indicated that activation of the PPP by pro-inflammatory cytokines caused a pro-oxidant environment by creating a situation in which free radical formation exceeds the capacity of the cell to generate GSH to increased vascular inflammation and induced the vascular damage 37. In addition, the PPP was inhibited by AGEs, but AGEs accelerated VC in VSMC 38. Although the HDHCP group had a lower mean value of AGEs compared to the HD group and slightly influenced VC, the activation of the PPP was contributed to developing higher calcium-phosphorus product.

The steroid biosynthesis pathway and the terpenoid backbone biosynthesis pathway were two increased lipid metabolic pathways in HDHCP. The terpenoid backbone biosynthesis produces steroid, and steroid and was converted to form cholesterol by the steroid biosynthesis pathway. Cholesterol produced cholesteryl esters by the esterification of cholesterol with long-chain fatty acids. Cholesterol ester will interact with triacylglycerol to produce very low-density lipoprotein (VLDL). In nondiabetic subjects, higher VLDL size was significantly associated with CAC 39. In a high-risk type-2 diabetic population, very low-density lipoprotein cholesterol (VLDL-C) levels were positively associated with increasing CAC 40. The VLDL-C level is an independent risk factor for all-cause and cardiovascular mortality in peritoneal dialysis patients 41. The elevated VLDL-C was significantly associated with elevated coronary heart disease risk in a large Chinese cohort study 42.

In addition, the fatty acid elongation pathway also enriched in HDHCP. The fatty acid elongation pathway produced palmitic acid (PA) which was the most abundant long-chain saturated fatty acid (LCFA) in plasma. PA was quantitatively the major fatty acid produced during hepatic lipogenesis to generate VLDL 43. PA induced osteoblastic differentiation and calcium deposition in VSMC through increasing the expression of the genes for bone-related proteins in human aortic smooth muscle cells and then inducing the activation of NF-kB 44. Long-chain fatty acids activated calcium channels and increased intracellular calcium in ventricular myocytes 45. The accumulation of LCFAs in mature erythrocytes was sensitive to single HD treatment which increased the cardiovascular risk in ESRD patients 46.

Our study demonstrated that *Lachnospiraceae_FCS020_group* was significantly increased in hemodialysis patients with VC compared to hemodialysis patients without VC. Perhaps *Lachnospiraceae_FCS020_group* was a potential biomarker for VC diagnosis. Besides, we found the changes of metabolic functions in hemodialysis patients with VC and the changes were related to causing VC. The clinical characteristics showed that Ca, Ca×P and iPTH of blood were significantly increased in hemodialysis patients with VC. Ca, P and iPTH are crucial to the simultaneous control of the mortality risk and cardiovascular hospitalization in dialysis patients 47-49. Differences of the gut microbiome, microbial metabolism and several clinical characteristics between two hemodialysis groups were probably factors to cause VC, including the gut microbiome, microbial metabolism and several clinical characteristics.

Overall, the gut microbiota appears to play a significant role in the development of VC in patients with CKD. Although the prediction of the functional profile using PICRUSt is sufficiently linked to the phylogeny and provides useful insights, it is not a direct evidence 24. Further research is needed to characterize the specific mechanisms through which the gut microbiota contributes to VC and to identify potential therapeutic strategies that may target these mechanisms to prevent or reverse VC in patients with CKD.

## Supplementary Material

Supplementary data.

## Figures and Tables

**Table 1:**
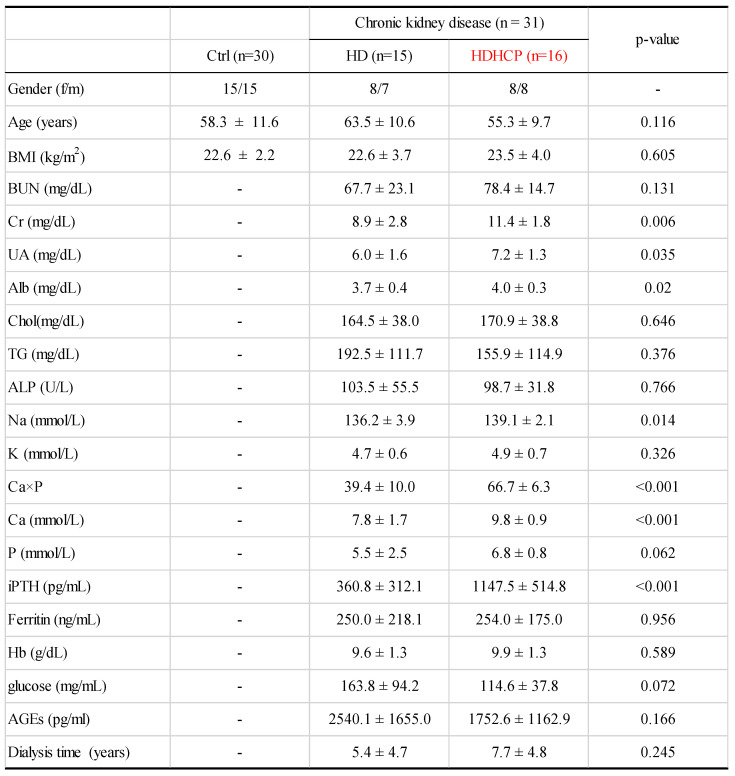
Clinical characteristics in this study were exhibited by patients with healthy controls (Ctrl), hemodialysis dialysis patients without higher Ca x P (HD), and hemodialysis patients with higher Ca x P (HDHCP). Categorical variables were compared using one-way ANOVA or Student's t-test.

**Table 2:** According to LEfSe results, 20 genera were significantly enriched in HDHCP.

**Table 3:** The canonical correspondence analysis of the associations between the gut microbiome and clinical indicators for hemodialysis patients.
