# Supplementary material for: Role of the gut microbiota and their metabolites in hemodialysis patients
Source: Int J Med Sci. 2023 Apr 17;20(6):725–36. doi: 10.7150/ijms.82667 (PMC10198149; doi:10.7150/ijms.82667)
Supplement: Supplementary file 1 — Supplementary data. [file ijmsv20p0725s1.pdf]

Supplementary data S1. Gut microbial diversity of healthy controls and hemodialysis patients. (A) Taxon richness and evenness in hemodialysis patients were significantly higher than healthy controls, Chao1 (B), Ace (C) and Shannon (D) diversity under healthy controls and hemodialysis patients. Taxon richness and evenness reveal the significance among HD and HDHCP groups ($p \leqq 0.0001$).

Supplementary data S2. Gut microbial alterations in health controls and hemodialysis patients.

Supplementary data S3. Phylum and genus level taxonomical succession of the gut microbiome in healthy controls and dialysis patients.

Supplementary data S1

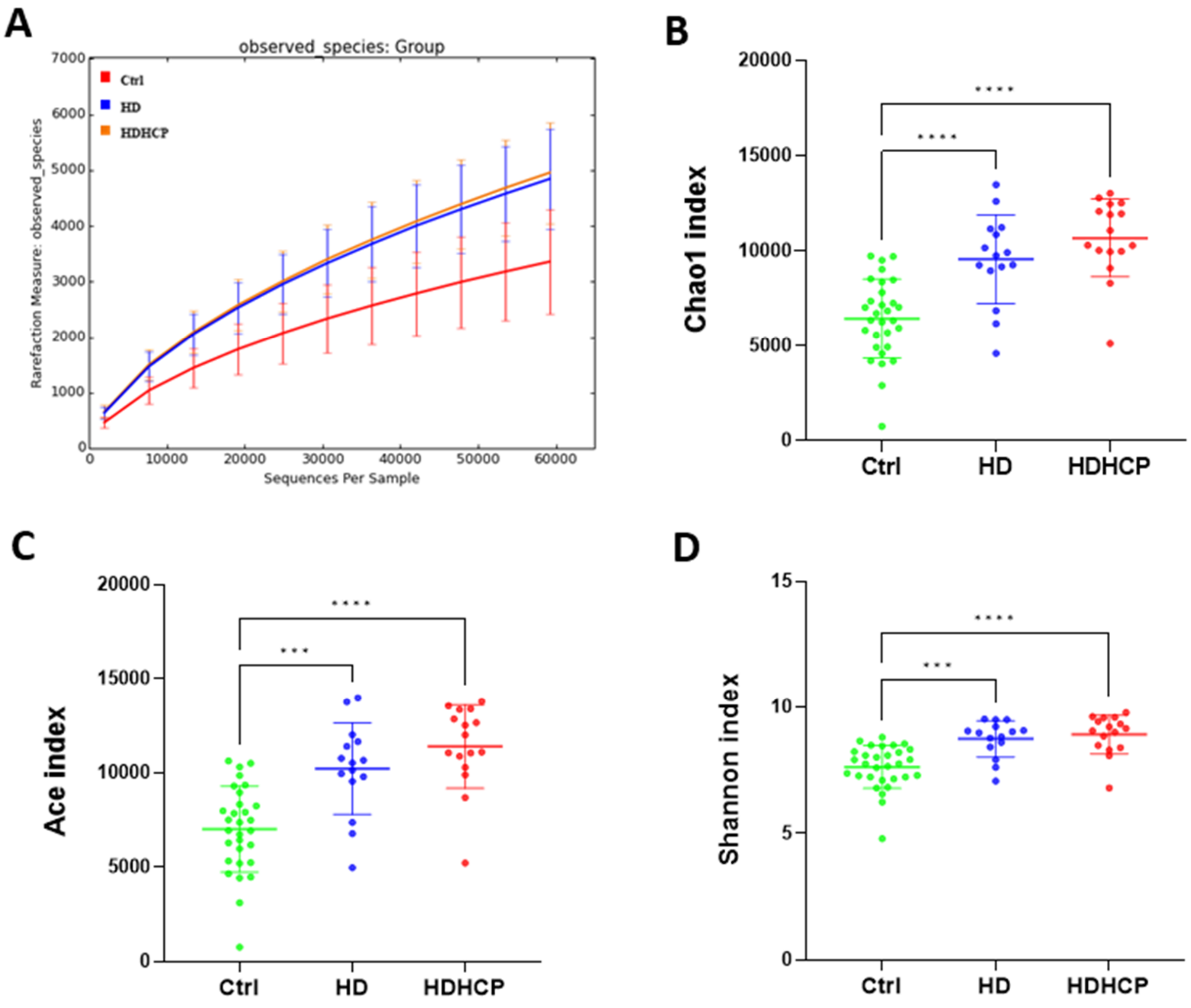

Supplementary data S2

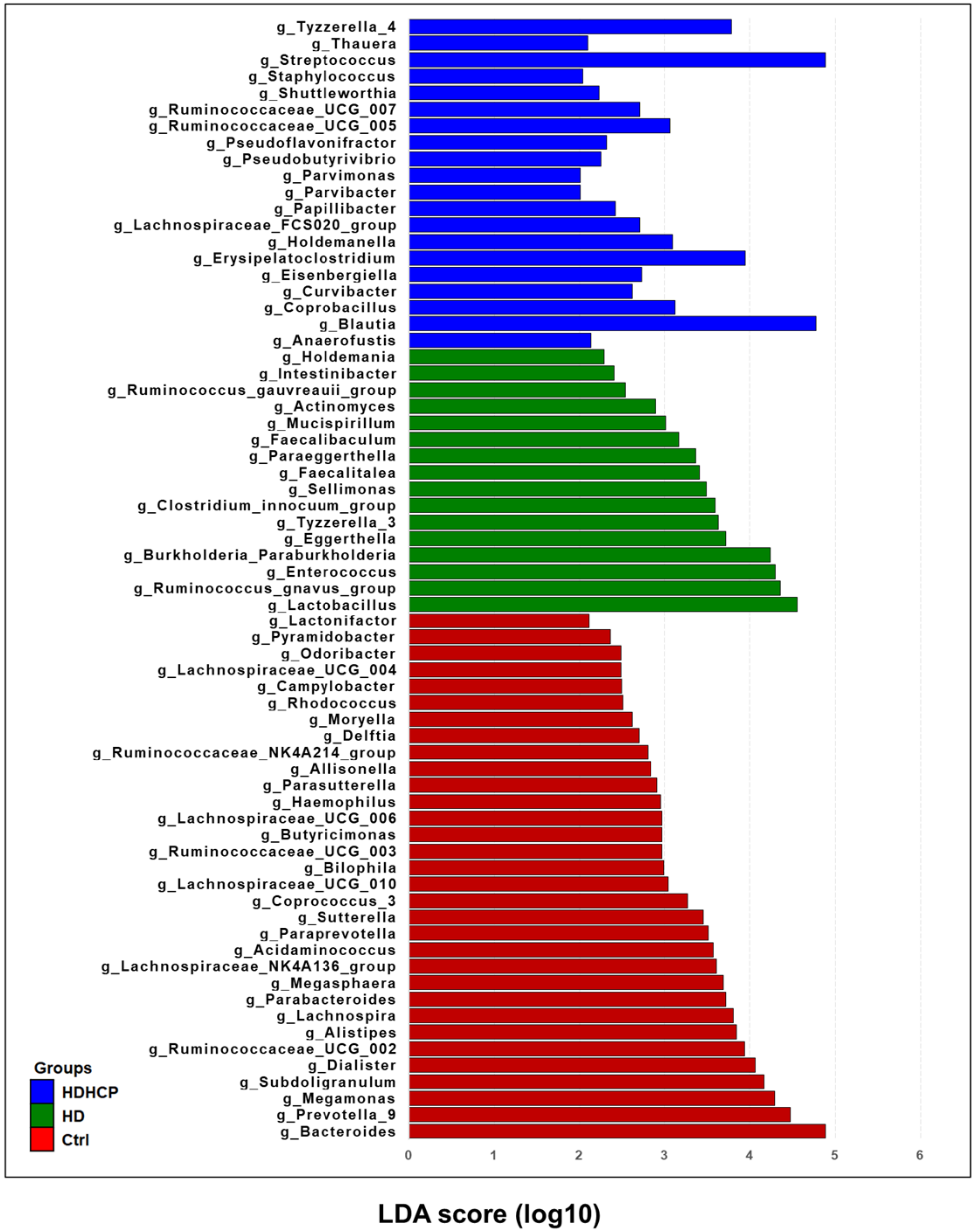

Supplementary data S3

Phylum	Ctrl	HD	HDHCP
Firmicutes	0.58	0.761	0.762
Actinobacteria	0.052	0.081	0.103
Bacteroidetes	0.312	0.055	0.067
Proteobacteria	0.036	0.097	0.063
Verrucomicrobia	0.011	0.002	0.004
Bacteria_unclassified	0.001	0.002	0.001
Fusobacteria	0.003	0.001	0
Euryarchaeota	0.002	0	0
Others	0.003	0.001	0

Genus	Ctrl	HD	HDHCP
Bacteroides	0.19	0.046	0.051
Blautia	0.036	0.144	0.146
Streptococcus	0.004	0.079	0.161
Lachnospiraceae_unclassified	0.031	0.049	0.038
Prevotella_9	0.07	0	0.005
Faecalibacterium	0.051	0.022	0.015
Bifidobacterium	0.03	0.024	0.04
Ruminococcus_gnavus_group	0.003	0.056	0.047
Collinsella	0.013	0.032	0.036
Clostridium_sensu_stricto_1	0.012	0.051	0.016
Megamonas	0.043	0	0
Subdoligranulum	0.035	0.01	0.006
Ruminococcus_2	0.015	0.017	0.03
Burkholderia-Paraburkholderia	0	0.038	0.034
Lactobacillus	0	0.053	0.013
Phascolarctobacterium	0.032	0.002	0.001
Enterococcus	0.003	0.051	0.003
Enterobacteriaceae_unclassified	0.006	0.037	0.01
Romboutsia	0.015	0.012	0.017
Dorea	0.016	0.007	0.015
Eubacterium_hallii_group	0.014	0.006	0.017
Dialister	0.024	0	0
Fusicatenibacter	0.008	0.009	0.021
Ruminococcaceae_UCG-002	0.02	0.002	0.003
Anaerostipes	0.009	0.007	0.015
Others	0.319	0.246	0.261
